# Plasmonic Nanoparticles with Quantitatively Controlled Bioconjugation for Photoacoustic Imaging of Live Cancer Cells

**DOI:** 10.1002/advs.201600237

**Published:** 2016-09-07

**Authors:** Chao Tian, Wei Qian, Xia Shao, Zhixing Xie, Xu Cheng, Shengchun Liu, Qian Cheng, Bing Liu, Xueding Wang

**Affiliations:** ^1^ Department of Biomedical EngineeringUniversity of MichiganAnn ArborMI48109USA; ^2^ IMRA America, IncAnn ArborMI48105USA; ^3^ Department of RadiologyUniversity of MichiganAnn ArborMI48109USA; ^4^ Department of UrologyUniversity of MichiganAnn ArborMI48109USA; ^5^ College of Physical Science and TechnologyHeilongjiang UniversityHarbin150080China; ^6^ Institute of AcousticsTongji UniversityShanghai200092China

**Keywords:** cancer cells, plasmonic nanoparticles, photoacoustic imaging, quantitative bioconjugation

## Abstract

Detection and imaging of single cancer cells is critical for cancer diagnosis and understanding of cellular dynamics. Photoacoustic imaging (PAI) provides a potential tool for the study of cancer cell dynamics, but faces the challenge that most cancer cells lack sufficient endogenous contrast. Here, a type of colloidal gold nanoparticles (AuNPs) are physically fabricated and are precisely functionalized with quantitative amounts of functional ligands (i.e., polyethyleneglycol (PEG) and (Arginine(R)–Glycine(G)–Aspartic(D))_4_ (RGD) peptides) to serve as an exogenous contrast agent for PAI of single cells. The functionalized AuNPs, with a fixed number of PEG but different RGD densities, are delivered into human prostate cancer cells. Radioactivity and photoacoustic analyses show that, although cellular uptake efficiency of the AuNPs linearly increases along with RGD density, photoacoustic signal generation efficiency does not and only maximize at a moderate RGD density. The functionalization of the AuNPs is in turn optimized based on the experimental finding, and single cancer cells are imaged using a custom photoacoustic microscopy with high‐resolution. The quantitatively functionalized AuNPs together with the high‐resolution PAI system provide a unique platform for the detection and imaging of single cancer cells, and may impact not only basic science but also clinical diagnostics on a range of cancers.

## 1 Introduction

Cancer is a group of fatal diseases that involve abnormal cell proliferation with the potential to invade and spread to other parts of the body.^1^ Early imaging and diagnosis of premalignant and malignant human cancers is essential for improving the survival rate of patients. Photoacoustic imaging (PAI), which is based on the conversion of light into sound, is an emerging, hybrid, and noninvasive biomedical imaging modality, and has been extensively explored for its applications in cancer detection and imaging.^2^, ^3^, ^4^, ^5^, ^6^ PAI has the advantages of high optical contrast and good ultrasound penetration, and can provide multidimensional tumor data, including structural, functional, metabolic, and molecular information.^7^ To date, most work has been focused on solid tumor detection at the macroscopic level.^7^, ^8^, ^9^, ^10^, ^11^, ^12^, ^13^, ^14^, ^15^, ^16^ However, solid tumor usually evolves from a single cell.^17^ When the number of cancerous cells does not reach a certain threshold (e.g., 10^9^),^17^ early tumors may be difficult to be detected, which limits PAI from early cancer detection and research of cellular dynamics.^17^, ^18^ From this standpoint, microscopic imaging of single or small numbers of cancer cells is highly desirable and of special significance.^19^, ^20^ This, however, imposes demanding requirements on the resolution and sensitivity of PAI because most cancer cells are very small in size (≈10 μm) and exhibit little endogenous absorption contrast (except melanoma cells).^21^, ^22^, ^23^, ^24^, ^25^, ^26^ To achieve high‐sensitivity PAI at the cellular level, exogenous contrast agent is preferably used to enhance optical absorption and amplify photoacoustic signal generation efficiency.^27^, ^28^, ^29^

Plasmonic gold nanoparticles (AuNPs), the best characterized and most widely used nanomaterial to date, are ideal contrast enhancers for photoacoustic imaging due to their excellent biocompatibility, low cytotoxicity, and superb optical absorptions up to five orders of magnitude higher than those of traditional organic dyes.^20^, ^29^, ^30^, ^31^, ^32^, ^33^ AuNPs have already been used in PAI to image and diagnose a variety of cancers,^7^, ^9^, ^10^, ^11^, ^12^, ^13^, ^14^, ^15^, ^16^ such as melanoma,^9^ squamous cell carcinoma,^10^ prostate cancer,^11^ colon carcinoma,^12^ breast cancer,^13^ and circulating tumor cells.^14^, ^15^, ^16^ Several parameters, such as size, shape, and surface chemistry, are critical for their photoacoustic applications.^34^ While the size and shape determine the optical properties, the surface chemistry controls the interactions of AuNPs with surrounding biological environments.^30^, ^35^ To achieve highly efficient delivery of AuNPs into cancer cells, active surface coating with functional ligands, such as hydrophilic polymers and targeting molecules, are required. Various strategies, including physical adsorption,^36^ thiolate chemisorption,^37^ and covalent coupling,^38^ have been developed to manipulate and modify AuNP surface chemistry. However, most methods are inadequate to achieve efficient and controllable functionalization of individual AuNPs, e.g., conjugating each AuNP with multiple types of ligands with precisely controlled amounts and distributions.^39^ Controllable functionalization to evaluate and optimize nanoparticle performances is important for many practical applications, such as multi‐type cancer cells targeting and efficient drug delivery.^40^ Achieving controllable functionalization of AuNPs with quantitative amounts of multi‐type ligands, although extremely important, is still a great challenge.

To address the problems of high‐sensitivity detection and high‐resolution imaging of single or small populations of cancer cells, we physically produced a kind of colloidal AuNPs with bare surfaces using ultrafast laser ablation of a gold target immersed in deionized water, precisely coated them with quantitative amounts of two different functional ligands, i.e., thiol‐terminated polyethylene glycol (PEG) molecules and cysteine‐modified (Arginine(R)–Glycine(G)–Aspartic(D))_4_ (RGD) peptides, and selectively delivered them into human prostate cancer cells to serve as an exogenous absorption contrast. Through quantitative bioconjugation, we were able to study the cellular uptake efficiency of the functionalized AuNPs (fAuNPs) using radioactivity analysis and their photoacoustic signal generation efficiency using photoacoustic analysis at different surface RGD densities. Based on the experimental results, we determined the optimal RGD ligand density that yields the highest photoacoustic signal amplitude, and imaged individual cancer cells using a custom high‐resolution photoacoustic microscopy (PAM) system.

## 2 Results and Discussion

### 2.1 Synthesis of the fAuNPs with Quantitatively Controlled Bioconjugation and Targeted Delivery into Human Cancer Cells

**Figure 1:**
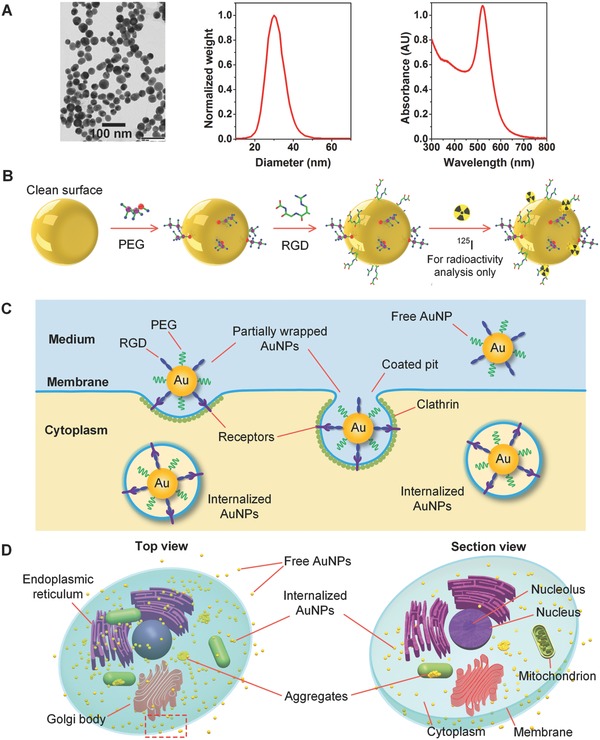
Physically generated colloidal AuNPs, functionalization and cellular uptake. A) TEM image, size distribution, and UV–vis absorption spectrum of the nanoparticles. B) Sequential conjugation with quantitatively controlled PEG molecules and RGD peptides, and radiolabeling with ^125^I radionuclide (for radioactivity analysis only). C) A schematic showing cellular uptake of the AuNPs via receptor‐mediated endocytosis. D) Top view (left) and section view (right) of a cancer cell with internalized AuNPs.

We first fabricated raw AuNPs using our patented technology,^41^ i.e., femtosecond laser ablation of a gold target submerged in flowing deionized water (see the Experimental Section and Figure S1 in the Supporting Information). This method uses tightly focused micro‐joule (μJ) femtosecond laser pulses to produce nanoparticles, and the size and size distribution of generated nanoparticles can be precisely controlled by optimizing laser parameters, such as wavelength, pulse energy, duration, and repetition rate, etc.^42^ Colloidal AuNPs with an average diameter of 30 nm were produced and used in our experiments. Representative transmission electron microscopy (TEM) image, hydrodynamic particle size distribution and ultraviolet–visible (UV–vis) absorption spectrum are presented in **Figure** **1**A. The generated nanoparticles have a narrow size distribution and have an absorption peak at 520 nm due to localized surface plasmon resonance (LSPR).^43^ The spectral feature below 450 nm reflects gold intraband transitions since the nanoparticles were generated in deionized water. Since no other chemical components, such as chemical precursors, reducing agents, and stabilizing ligands, are involved in the fabrication process, the colloidal AuNPs produced using the laser ablation method are naturally negatively charged and surfactant‐free compared with chemically synthesized nanoparticles.^35^ This unique feature allows versatile and controllable surface manipulation and modification, and serves as the foundation of quantitative bioconjugation of the colloidal AuNPs.

Following the methodology developed in our previous work,^35^ the raw colloidal AuNPs were quantitatively functionalized with two different types of ligands, i.e., PEG molecules and RGD peptides for effective and selective targeting of cancer cells (see the first two Steps in Figure 1B and the Experimental Section). The functionalization was performed in a sequential manner by first mixing the colloidal AuNPs with PEG and then RGD peptides, both of which are terminated with thiol groups (SH). The quantification was achieved by precisely controlling the molar ratio of the ligands to the colloidal AuNPs, i.e., PEG/AuNPs and RGD/AuNPs. PEG were grafted onto the nanoparticles because they can improve their stability and biocompatibility, and simultaneously minimize nonspecific interactions with biological tissues. RGD peptides were coated on the nanoparticles because they can specifically bind with complementary proteins (known as receptors) on plasma membrane of cancer cells to improve cellular recognition and uptake, which involves a complex succession of biomechanical and biochemical events: docking, membrane wrapping, pinching off, intracellular trafficking, etc. (Figure 1C). The binding of PEG molecules and RGD peptides to the nanoparticles is possible due to strong anchoring of the thiol‐gold bonds, which covalently attach the chains of the ligands to the surfaces of the nanoparticles. The binding as well as quantitatively controlled bioconjugation were experimentally confirmed by dynamic light scattering (DLS) measurements (Figures S2–S4 in the Supporting Information). The two types of grafted functional ligands allow the AuNPs to be stably present in cellular microenvironments, and be selectively internalized into cancer cells via receptor‐mediated endocytosis (Figure 1D). The targeted delivery and presence of the fAuNPs in human cancer cells was experimentally validated using TEM imaging (see the Experimental Section and Figure S5 in the Supporting Information).

### 2.2 Radioactivity Analysis of Cellular Uptake Efficiency of the fAuNPs at Different RGD Densities

**Figure 2:**
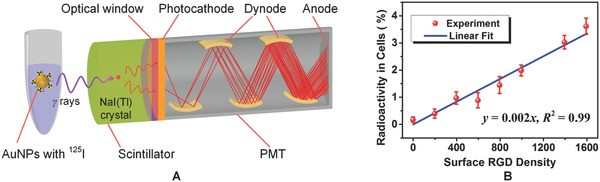
Evaluation of cellular uptake efficiency of the ^125^I‐labeled AuNPs at different surface RGD densities using radioactivity analysis. A) Schematic diagram showing how the gamma counter counts gamma radiations. B) Experimental results showing cellular uptake efficiency of AuNPs at different surface RGD densities.

Quantitative functionalization of the nanoparticles provides us a unique tool to precisely control and optimize the functionality of AuNPs for future clinical applications. For detection and imaging of single or small numbers of cancer cells, the cancer‐targeting RGD ligand plays a critical role because its density has a direct impact on the cellular uptake efficiency and the average number of AuNPs internalized into per cell. This in turn is very important for preclinical testing of nanoparticles to evaluate dose‐efficacy relationships and optimize biophysical and biochemical parameters.^44^, ^45^ A series of fAuNP solutions with a fixed PEG density (PEG/AuNPs = 450) but varying RGD densities (RGD/AuNPs from 200 to 1600) were prepared in the experiment. We then studied the dependence of cellular uptake efficiency of the fAuNPs on the surface RGD density using radioactivity analysis.^46^, ^47^ By employing a gamma counter, the technique enables us to quantitatively measure gamma radiation from radionuclides immobilized on the AuNPs (**Figure** **2**A and the Experimental Section).

Due to the need of radionuclides in radioactivity analysis, the synthesized fAuNPs at different RGD densities were further radiolabeled with ^125^I radioisotope by simply mixing them with ^125^I sodium iodide solutions (see Step 3 in Figure 1B and the Experimental Section). This radiolabeling procedure has been proven to be efficient and stable as a result of the high affinity and strong binding of iodide ions with the surface of AuNPs.^27^ In the experiment, the ^125^I‐labeled fAuNPs were delivered into human prostate cancer cells (DU145) for quantitative evaluation of cellular uptake efficiency. The treated cancer cells were finally separated from culture media and washed with fresh phosphate buffered saline (PBS) for radioactivity analysis. All the three parts, i.e., cells, culture media, and PBS, contained ^125^I radionuclides. Radioactivity of each part was measured and recorded using a gamma counter. The percentage of radioactivity remaining in the cells was calculated to reflect the cellular uptake efficiency at different RGD densities (see the Experimental Section).

The measured cellular uptake efficiency at different RGD densities are shown in Figure 2B (see Table S1 in the Supporting Information for raw data). Two conclusions can be drawn from the experimental results. First, cellular uptake of the fAuNPs is a specific targeting process, during which the RGD peptides play a decisive role. Without the RGD ligands, only a very limited number of nanoparticles could enter the cells (see the control data at RGD 0 in Figure 2B). Second, cellular uptake efficiency of the fAuNPs has a strong linear correlation with the surface RGD density within the range of 200 to 1600. This suggests that, in practical applications, we can regulate the amount of AuNPs delivered into each cancer cell by tuning the surface RGD density. Moreover, the results also indirectly support our quantitatively controlled bioconjugation claim in the previous Section, because cellular uptake efficiency markedly differs at different RGD densities. The radioactivity analysis provides us important information to quantitatively understand cellular uptake efficiency of AuNPs at different surface RGD densities.

### 2.3 Photoacoustic Analysis of Acoustic Signal Generation Efficiency of the fAuNPs at Different RGD Densities

**Figure 3:**
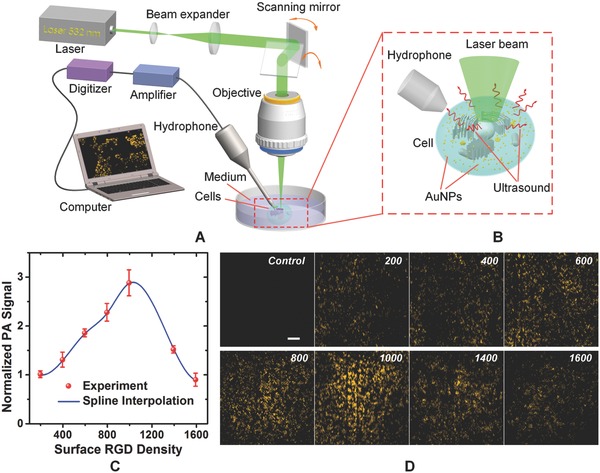
Evaluation of photoacoustic signal generation efficiency of intracellular AuNPs at different surface RGD densities using photoacoustic analysis. A,B) Schematic representation of the laser‐scanning PAM and enlarged view of the sample part in the red dashed box. C) Normalized cumulative signal intensities of 2D photoacoustic images at different RGD densities (each data point with five independent measurements). The results show photoacoustic signal generation efficiency first rises with the increase of RGD density and then declines after reaching the maximum at RGD 1000. D) Typical PAM images of intracellular AuNPs at different RGD densities. Data are presented as means ± SEM (standard deviation of the mean). Scale bar: 100 μm.

The radioactivity analysis results show that cellular uptake of AuNPs increases linearly with the surface RGD density between 200 and 1600. Since the functionalized nanoparticles are designed for PAI, we further investigated the dependence of photoacoustic signal generation efficiency of intracellular AuNPs on the surface RGD density using our custom laser‐scanning PAM (see **Figure** **3**A,B and the Experimental Section). The PAM system employs a 532‐nm nanosecond laser as the illumination source to excite the samples, a 2D (*x* and *y*) galvanometer as the scanning mechanism to control the angle of the deflected beam, an achromatic lens as the objective to focus the laser beam, and a hydrophone as the transducer to receive excited photoacoustic waves. The PAM has a calibrated lateral resolution of 1.6 μm, which is sufficient for identifying single cells, such as cancer cells and red blood cells (average diameter 7.2 μm,^48^ see Note S2 in the Supporting Information). The photoacoustic signal *p* generated from intracellular AuNPs at different RGD densities ρ_RGD_ can be written as

(1) $$p=\Gamma F\mu_{a}(\rho_{\mathrm{RGD}})$$

where Γ is the Grüneisen parameter (constant for a particular sample), *F* is the laser fluence (fixed throughout the experiment), and μ_a_ is the optical absorption coefficient at 532 nm. The photoacoustic signal *p* is linearly proportional to the absorption coefficient μ_a_, which is a function of the surface RGD density ρ_RGD_.

In the experiment, fAuNP solutions with different surface RGD densities were delivered into different groups of DU145 cells, which were cultured in petri dishes and had a near 100% confluence (see the Experimental Section). Each group was randomly imaged using the PAM at five different regions (*n* = 5), each with a field of view of 1 mm × 1 mm under the same conditions. All photoacoustic signals within each image were summed up and the average of the five tests was used to represent the corresponding photoacoustic signal generation efficiency at a particular RGD density. The normalized results with respect to the value at RGD 200 are shown in Figure 3C. It shows that the photoacoustic signal generation efficiency of intracellular nanoparticles would first rise with the increase of RGD density, and then decline after reaching the maximum at RGD 1000. The result is different from that of the radioactivity analysis, but they are not necessarily contradictory because they reflect two different processes. Specifically, radioactivity analysis represents intracellular uptake efficiency of nanoparticles at different RGD densities and is essentially a biological process, while photoacoustic analysis indicates signal generation efficiency of intracellular nanoparticles and is essentially a physical process. A higher amount of cellular uptake of the nanoparticles does not necessarily lead to greater photoacoustic signals when aggregation happens to the internalized nanoparticles, which was confirmed by the TEM imaging result (see Figure S5 in the Supporting Information). The optical absorption μ_a_ of the AuNPs depends on the LSPR, or the resonant oscillation of conduction electrons when stimulated by light. When the nanoparticles aggregate, the conduction electrons near each particle surface will be delocalized and shared amongst neighboring particles, which shifts the LSPR to lower energies and absorption peak to longer wavelength. The redshift leads to a drop in optical absorption μ_a_ at 532 nm, and, therefore, lower photoacoustic signals [Equation (1)]. This finding is of great importance for the optimal design of plasmonic nanoparticles for absorption‐based biomedical applications, such as photothermal therapy and PAI. Figure 3D shows representative PAM images of intracellular AuNPs at different RGD densities, which reveals that RGD 1000 gives the highest photoacoustic signals.

### 2.4 Photoacoustic Imaging of Live Human Cancer Cells Enhanced by the fAuNPs with Optimal Signal Generation Efficiency

The radioactivity analysis of cellular uptake efficiency and photoacoustic analysis of signal generation efficiency provides a very important foundation on optimizing the performance of the nanoparticles. For PAI of single or small numbers of cancer cells, we prefer maximizing the photoacoustic signal generation efficiency of intracellular nanoparticles to achieve high‐sensitivity imaging. Using the experimentally obtained optimal RGD density 1000, we carried out one more experiment to demonstrate high‐sensitivity detection and high‐resolution imaging of small numbers of cancer cells using the custom PAM. In this experiment, the fAuNPs with a RGD density 1000 at a concentration of 200 × 10^−12^ m was delivered into DU145 cancer cells (see the Experimental Section). Treated cells with an about 50% confluence were used for imaging.

**Figure 4:**
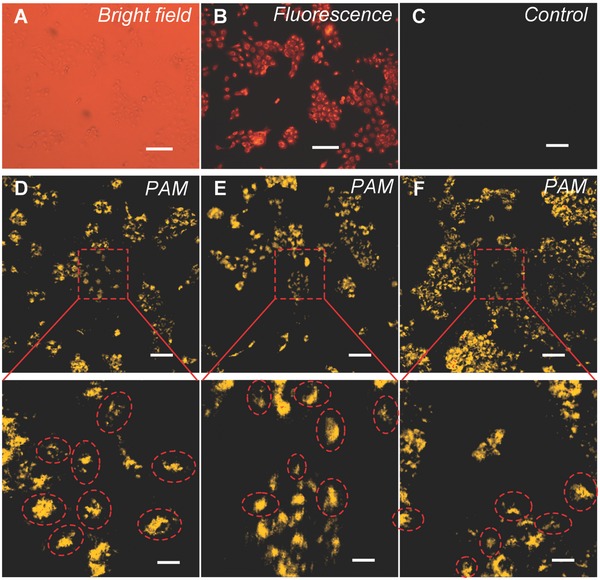
Detection and imaging of single human prostate cancer cells. A,B) Bright‐field and co‐registered fluorescence microscopy images. C) Control image showing no noticeable photoacoustic signals were observed for cells not treated with the fAuNPs. D–F) Three representative PAM images and enlarged views. Single cancer cells in dashed circles are visible and discernable in the PAM images. Scale bar: 100 μm (25 μm in the close‐up views).

The presence of the live cancer cells in the dish was first confirmed by bright‐field and fluorescence imaging using the same optical microscope (see the Experimental Section for cell staining and fluorescence imaging). The coregistered bright‐field and fluorescence images, presented in **Figure** **4**A,B, show typical morphology and distribution of the DU145 cells. The cell samples were then transferred to the custom PAM system for imaging. No noticeable photoacoustic signals were observed in control cells not treated with the fAuNPs, as shown in Figure 4C. In contrast, in cells incubated with the fAuNPs, strong photoacoustic signals were produced and captured by the hydrophone. High resolution 2D photoacoustic images were delineated by scanning the galvanometer. Three representative PAM images and their enlarged views, showing cell distribution and morphology, are presented in Figure 4D–F. Single live DU145 cells (≈20 μm in diameter) are clearly visible and easily discernible in the images, as marked by the dashed circles. The quantified signal‐to‐noise ratio and contrast‐to‐noise ratio of all images are greater than 17.0 dB (see Note S1 in the Supporting Information), which indicates excellent image quality. Although the PAM images are not coregistered with the fluorescence image, the morphology and distribution of the cells look similar to those in the fluorescence image, which indirectly supports the validity of the photoacoustic results.

## 3 Conclusion

We demonstrated high‐sensitivity and high‐resolution PAI of single and small populations of live human cancer cells using quantitatively functionalized gold nanoparticles (fAuNPs) as exogenous contrast agent. The fAuNPs were synthesized by coating colloidal AuNPs with two different types of functional ligands, i.e., PEG molecules and RGD peptides, in precisely controlled amounts. The colloidal AuNPs were physically fabricated by ultrafast laser ablation of a bulk gold target in flowing deionized water and have the advantages of being naturally negatively charged and surfactant‐free. This unique feature allows the nanoparticles to be versatilely and controllably engineered with different types and precise amounts of functional ligands, and serves as the basis of the quantitative bioconjugation.

The fAuNPs with different surface RGD densities were selectively delivered into human prostate cancer cells through receptor‐mediated endocytosis. Radioactivity analysis shows that cellular uptake efficiency, or the number of the nanoparticles internalized into each cell, rises linearly when surface RGD density increases from 200 to 1600. Photoacoustic analysis reveals that photoacoustic signal generation efficiency first rises with the increase of RGD density, but then declines after reaching the maximum at RGD 1000. The decrease in photoacoustic signal generation efficiency reflects intracellular nanoparticle aggregation and the resulting redshift in optical absorption. This experimental finding helps to determine the optimal surface RGD density for achieving high‐sensitivity microscopic PAI of live human cancer cells. The platform integrating the quantitatively bioconjugated AuNPs with high‐resolution and high‐sensitivity PAI may shed new light on cellular imaging, molecular imaging, clinical diagnostics, and optimized cancer therapy.

## 4 Experimental Section

*Sample Preparation*: All chemicals were used as received without further purification. Thiol‐terminated PEG (PEG‐SH) with a molar mass of 5000 g mol^−1^ was purchased from Creative PEGWorks (Chapel Hill, NC, catalog # PLS‐604). Cysteine‐modified (RGD)_4_ peptides (molar mass: 1845.98 g mol^−1^, purity: >95%) with an amino acid sequence RGDRGDRGDRGDPGC were custom‐synthesized by RS synthesis LLC (Louisville, KY). PEG and RGD peptides were in powder form and dissolved in deionized water having an electric conductivity less than 0.7 μS cm^−1^. All solutions were freshly made as needed and used within twelve hours.

*Physical Production and Characterization of the Colloidal AuNPs*: Colloidal AuNPs with bare surfaces were physically fabricated using the patented technology,^41^, ^42^ i.e., femtosecond laser ablation of a bulk gold target (16 mm long, 8 mm wide, 0.5 mm thick, and 99.99% purity, Alfa Aesar, Ward Hill, MA) in flowing deionized water (see Figure S1 in the Supporting Information). The ytterbium‐doped femtosecond fiber laser (FCPA μJewel D‐1000, IMRA America, Ann Arbor, MI) operating at 1.045 μm delivered pulsed laser at a repetition rate of 100 kHz with 10 μJ pulse energy and 700 fs pulse duration. The emitted laser beam was first focused by an objective lens, and then reflected by a scanning mirror to the surface of the bulk gold target, which was submerged in flowing deionized water (18 MΩcm). The size of the laser spot on the gold target was estimated to be 50 μm and its position was precisely controlled by the scanning mirror. During the ablation process, a translation stage was employed to produce relative movements between the laser beam and the gold sample.^41^, ^42^ The generated nanocolloids were stably suspended in water and did not require any dispersants, surfactants or stabilizers to maintain their stability. This unique advantage allows production of colloidal AuNPs with bare surfaces, which facilitates subsequent surface modification process.

The produced colloidal AuNPs were characterized by an array of analytic instruments and techniques, including TEM, DLS, and UV–vis) spectroscopy. Images of the colloidal AuNPs were recorded using a TEM (JEOL 2010F, Japan) at an accelerating voltage of 100 kV. Nanoparticle hydrodynamic diameter size and zeta potential were characterized via DLS analyses using a Nano‐ZS90 Zetasizer (Malvern Instrument, Westborough, MA). UV–vis absorption spectra were recorded by a spectrophotometer (UV‐3600, Shimadzu Corp., Japan).

*Quantitatively Controlled Bioconjugation of the Colloidal AuNPs*: A sequential conjugation procedure was followed to graft quantitative PEG molecules and RGD peptides onto the surface of the colloidal AuNPs.^35^ The functionalization process comprises two steps.

Step 1. PEGylated the colloidal AuNPs (average diameter 30 nm, optical density or OD 1) by mixing it with the PEG‐SH solution. The molar ratio of the PEG solution to the colloidal AuNPs was tuned to 450 (i.e., PEG/AuNPs = 450) to keep the stability of the nanoparticles, and, at the same time, avoid excessive surface coverage (see Figure 1B). In this way, enough surface space will be left on the surface of the AuNPs for subsequent RGD peptides conjugation. The mixture was allowed to stand for two hours at room temperature to enable sufficient conjugation of PEG with the AuNPs via thiol‐gold bonding. The PEGylated AuNP solution was centrifuged (5000 g, 10 min) twice with supernatants discarded. The final PEGylated AuNPs were collected and resuspended in deionized water to an OD of 20. It is worth noticing that the conjugation of PEG molecules to the physically generated AuNPs is very efficient when the molar ratio between PEG molecules and AuNPs is below the saturation point according to our previous study.^49^ For this specific case, the saturation molar ratio is around 1000 (see Figure S2 in the Supporting Information), and the experimentally adopted molar ratio 450 is well below it, which indicates that the conjugation of PEG molecules onto AuNPs was complete in the experiment.

Step 2. Conjugated the PEGylated AuNPs (OD 20) with RGD peptides by mixing it with RGD peptides. The number of RGD peptides conjugated on the surface of the AuNPs was quantitatively determined by controlling the molar ratio of the RGD peptides to the AuNPs (e.g., RGD/AuNPs = 200, 400, 600, 800, 1000, 1400, and 1600 in the experiment). The resultant solutions were allowed to stand for another two hours at room temperature to ensure sufficient conjugation of RGD peptides onto unoccupied surface space of the AuNPs. The final solutions were again centrifuged (5000 g, 10 min) twice with supernatants removed. The resultant fAuNPs were collected and resuspended in borate buffer (pH 8.2) to an OD of 20 as the stock solution. It should be noted that real RGD density (number of RGD peptides per nanoparticle) and the molar ratio mentioned here are not exactly the same due to possible incomplete conjugation. But below the saturation molar ratio 1600 (see Figure S4 in the Supporting Information), more and more RGD ligands were expected to be conjugated onto the AuNPs with the increase of the molar ratio. Therefore, it makes sense to use the molar ratio to reflect the relative RGD density without affecting the final conclusion. In other words, the RGD density in this paper actually means the molar ratio between RGD peptides and AuNPs instead of absolute number of RGD peptides per nanoparticle.

The quantitatively controlled bioconjugation of the nanoparticles with PEG molecules and RGD peptides were confirmed via measuring their hydrodynamic diameters and zeta potentials by using DLS analyses (see Figures S2–S4 in the Supporting Information).

*Cell Culture and Treatment with the fAuNPs*: The human prostate carcinoma cell line (DU145, ATCC HTB‐81), culture medium (Eagle's minimum essential medium, EMEM, ATCC 30‐2003), fetal bovine serum (FBS, ATCC 30‐2020), and penicillin–streptomycin solution (ATCC 30‐2300) used in the experiments were all purchased from American Type Culture Collection (ATCC, Manassas, VA). The DU145 cancer cells were cultured in sterile petri dishes (9 cm in diameter) in EMEM supplemented with 10% FBS and 1% penicillin–streptomycin solution at 37 °C in a humidified atmosphere containing 5% CO_2_. The culture media were changed every other day.

At desired confluences, culture media were drained off from the dishes, and 2.7 mL FBS‐free EMEM and 0.3 mL fAuNPs with a concentration of 2 × 10^−9^ m (OD 6) were then added. The cells were again incubated at 37 °C in a humidified atmosphere containing 5% CO_2_. After culturing for twelve hours, the media and the nanoparticle solution were removed and the cells were rinsed with 3 mL 1× PBS buffer three times to wash off excess nanoparticles. The washed cells were immersed in fresh media and used for subsequent imaging experiments.

*Radiolabeling fAuNPs with ^125^I and Radioactivity Analysis*: Iodine‐125 (^125^I) radionuclide in 10^−5^ m NaOH (pH 8–11) was purchased from PerkinElmer (NEZ033A002MC) with a specific activity of 100 mCi mL^−1^. The [^125^I]NaI solution was first diluted to an activity of 0.5 mCi mL^−1^. The diluted solution (0.1 mL) was mixed with the fAuNPs solution (0.5 mL) and gently agitated for 10 min at room temperature. The mixture was then centrifuged at 7000 rpm for 15 min to remove unbound iodide and washed twice using Milli‐Q water (Merck Millipore, Billerica, MA). The ^125^I‐labeled fAuNPs were then resuspended in Milli‐Q water to a concentration of 2 × 10^−9^ m and measured using absorption spectroscopy at 520 nm. The radiolabeled fAuNPs were found to be stable for at least one week when stored in a refrigerator at 4 °C.

The radiolabeled fAuNP solution was then used to treat the DU145 cancer cells to study the cellular uptake efficiency of AuNPs. Specifically, 2.7 mL FBS‐free EMEM and 0.3 mL ^125^I‐labeled fAuNPs (2 × 10^−9^ m, OD 6) were added to the cell culture dishes after draining off previous culture media. The immersed cells were again incubated at 37 °C in a humidified atmosphere containing 5% CO_2_. After twelve hours in culture, the media containing non‐binding nanoparticles were removed and the cells were rinsed with 3 mL 1X PBS buffer three times to wash off excess nanoparticles. The washed cells were then treated with 3 mL 0.05% trypsin‐ethylenediaminetetraacetic acid, (Thermo Fisher Scientific, 25200056) solution for 10 min to ensure detachment from the bottoms of the culture dishes for subsequent radioactivity analysis.

Radioactivity was measured using a gamma counter (Packard Cobra II, GMI Inc., Ramsey, MN), which mainly consists three parts, i.e., a scintillator, a photomultiplier tube (PMT), and an optical window between them. It works as follows (see Figure 2A). When gamma rays radiated from the sample strike the scintillating crystal NaI(Tl), they will trigger the release of low‐energy photons, a portion of which pass through the optical window and reach the photocathode of the PMT. The photons are then converted into photoelectrons and multiplied in the PMT. The output signal of the PMT is proportional to the incident gamma radiation energy and measures the radioactivity in the samples. In this case, the ^125^I radionuclides exist in three different parts, i.e., cells, culture media, and PBS buffer used to wash the cells. The radioactivity of each part was separately measured and recorded using the gamma counter. A percentage, defined as the radioactivity remaining in the cells over the total amount of radioactivity in all three parts in each plate, was calculated to reflect the cellular uptake efficiency at different surface RGD densities. The percentage of radioactivity in cells *p*_cells_ is represented as

(2) $$p_{\mathrm{cells}}=\frac{\gamma_{\mathrm{cells}}}{\gamma_{\mathrm{cells}}+\gamma_{\mathrm{media}}+\gamma_{\mathrm{PBS}}}$$

where γ_cells_, γ_media_, and γ_PBS_ are the radioactivity (gamma signals) in cells, culture media, and PBS, respectively.

*TEM Imaging*: Prior to TEM imaging, the treated cells were fixed in 1 mL fresh 4% paraformaldehyde in 1X PBS for 15 min and followed by three washes with 5 mL 1X PBS buffer. Sections were cut and stained with aqueous uranyl acetate and lead citrate, and then mounted on a 300 mesh copper grid. The specimens were finally observed by TEM (JEOL 2010F, Japan) at an accelerating voltage of 100 kV.

*Fluorescence Imaging*: The stain (FilmTracer calcein red–orange biofilm stain, F10319) used in fluorescence imaging was purchased from Thermo Fisher Scientific. The stock staining solution was prepared by adding 50 μL dimethyl sulfoxide to 50 μg biofilm stain and mixed thoroughly until contents were completely dissolved. The working staining solution was prepared by diluting 10 μL stock solution with 990 μL 1X PBS buffer.

After draining off the culture media, 1 mL working solution was added to the dish and gently agitated to cover all cells. The cells were then incubated at 37 °C in a humidified atmosphere containing 5% CO_2_ for one hour to achieve uniform labeling. Afterward, the staining medium was drained off and the cells were washed three times. For each wash cycle, the cells were covered with 3 mL fresh PBS, incubated for 10 min, and then drained. The stained cells were finally immersed in fresh culture media and photographed using a Leica DMIL fluorescence microscope (Leica, Germany) with a 10×/0.3 objective.

*PAM Imaging of Live Cancer Cells*: The laser‐scanning PAM was a custom, in‐house system,^50^, ^51^ as shown in Figure 3A. The laser source is a diode‐pumped solid‐state Nd:YAG laser (Spot‐10‐200‐532, Elforlight Ltd., UK) with a wavelength of 532 nm and a pulse duration of 2 ns. The emitted laser was first collimated by a lens system, then reflected by a 2D (*x*, *y*) galvanometer (6230H, Cambridge Technology, Bedford, MA), and finally, focused on the sample by an achromatic objective (AC254‐040‐A, Thorlabs, Newton, NJ) with a focal length of 40 mm and a numerical aperture of 0.2. The cells, grown on the bottom of petri dish and immersed in culture media, absorbed laser energy and produced acoustic signals. The generated acoustic signals were captured by a customized hydrophone (center frequency: 35 MHz, 100% bandwidth at −6 dB), amplified by a low‐noise amplifier (ZFL‐500LN, Mini‐Circuits, Brooklyn, NY), digitized by an A/D card (Cobra CompuScope CS22G8, GaGe, Lockport, IL), transferred to the computer, and finally reconstructed using a maximum amplitude projection algorithm for visualization.

## Supporting information

As a service to our authors and readers, this journal provides supporting information supplied by the authors. Such materials are peer reviewed and may be re‐organized for online delivery, but are not copy‐edited or typeset. Technical support issues arising from supporting information (other than missing files) should be addressed to the authors.

Supplementary
