# Supplementary material for: Plasmonic Nanoparticles with Quantitatively Controlled Bioconjugation for Photoacoustic Imaging of Live Cancer Cells
Source: Adv Sci (Weinh). 2016 Sep 7;3(12):1600237. doi: 10.1002/advs.201600237 (PMC5157183; doi:10.1002/advs.201600237)
Supplement: Supplementary file 1 — Supplementary [file ADVS-3-0-s001.pdf]

Copyright WILEY-VCH Verlag GmbH & Co. KGaA, 69469 Weinheim, Germany, 2016.

ADVANCED SCIENCE Open Access

Supporting Information

for *Adv. Sci.,* DOI: 10.1002/advs.201600237

Plasmonic Nanoparticles with Quantitatively Controlled Bioconjugation for Photoacoustic Imaging of Live Cancer Cells

Chao Tian, Wei Qian, Xia Shao, Zhixing Xie, Xu Cheng, Shengchun Liu, Qian Cheng, Bing Liu, and Xueding Wang**

Supporting Information for

Plasmonic Nanoparticles with Quantitatively Controlled Bioconjugation for Photoacoustic Imaging of Live Cancer Cells

Chao Tian,[1] *Wei Qian,*[2,*] *Xia Shao,*[3] *Zhixing Xie,*[3] *Xu Cheng,*[4] *Shengchun Liu,*[5]

Qian Cheng,[6] *Bing Liu,*[2] *and Xueding Wang*[1,3,6,*]

[1] Department of Biomedical Engineering, University of Michigan, Ann Arbor, MI 48109, United States

[2] IMRA America, Inc., 1044 Woodridge Avenue, Ann Arbor, Michigan 48105, United States

[3] Department of Radiology, University of Michigan, Ann Arbor, MI 48109, United States

[4] Department of Urology, University of Michigan, Ann Arbor, MI 48109, United States

[5] College of Physical Science and Technology, Heilongjiang University, Harbin 150080, China

[6] Institute of Acoustics, Tongji University, Shanghai 200092, China

Table of Contents

Supporting Figures and Tables 2

Supporting Notes 6

Note S1: SNR and CNR 6

Note S2: Lateral Resolution Calibration of the PAM System 6

References 8

Supporting Figures and Tables

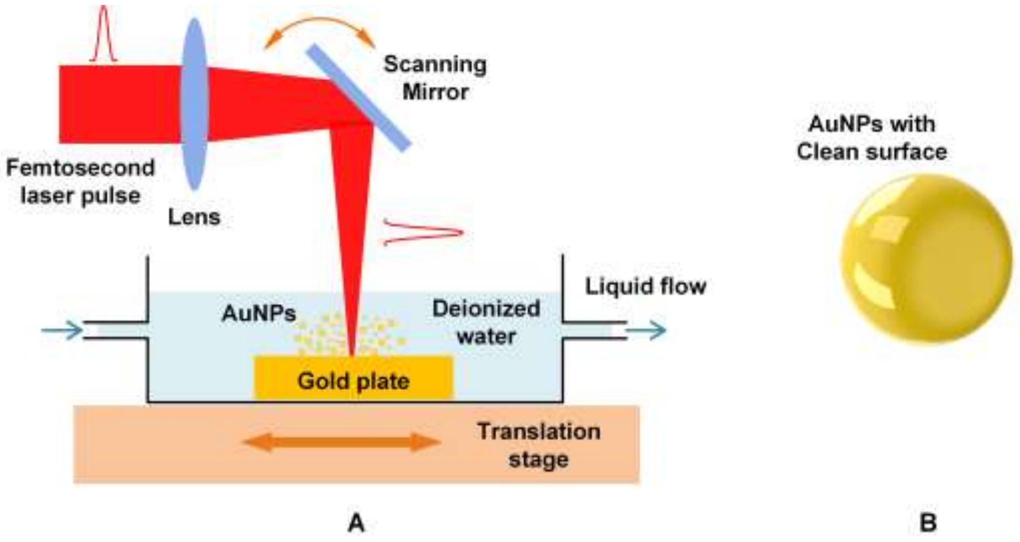

Figure S1. (**A**) Schematic setup illustrating the fabrication principle of the colloidal gold nanoparticles (AuNPs) using femtosecond laser ablation of a bulk gold target in flowing deionized water. (**B**) Schematic showing that produced AuNPs have clean surfaces.

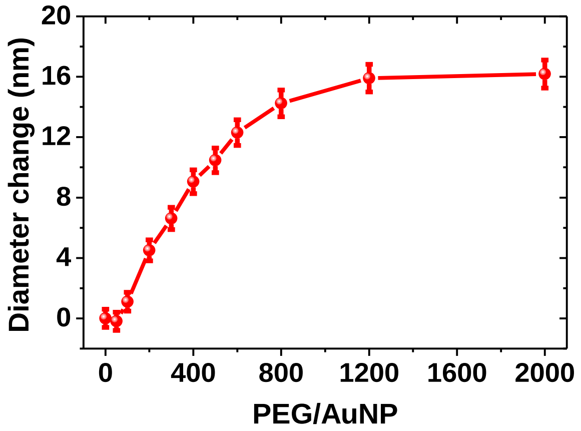

Figure S2. Diameter change of the colloidal AuNPs after being PEGylated with different amount of PEG measured by dynamic light scattering (DLS). PEG/AuNP represents the molar ratio of PEG to AuNP, and PEG/AuNP = 450 was used throughout the experiment to 1) keep the stability of the AuNPs, and 2) leave enough surface space for subsequent RGD peptides conjugation.

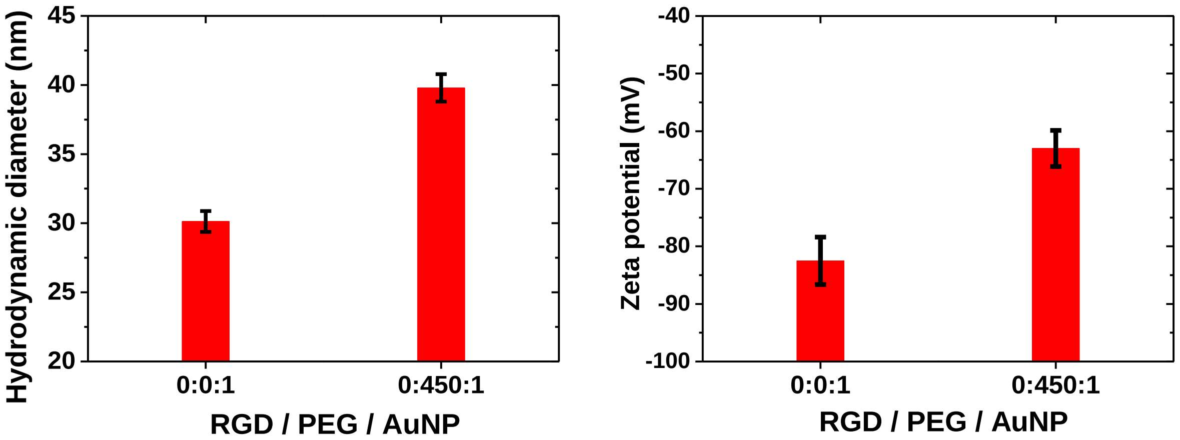

Figure S3. Hydrodynamic diameters (left) and zeta potentials (right) of the AuNPs before and after PEGlyation (the first step of the sequential conjugation). The results show that the diameter of the colloidal AuNPs increased by about 9 nm, and absolute zeta potential decreased by about 20 mV after PEGlyation.

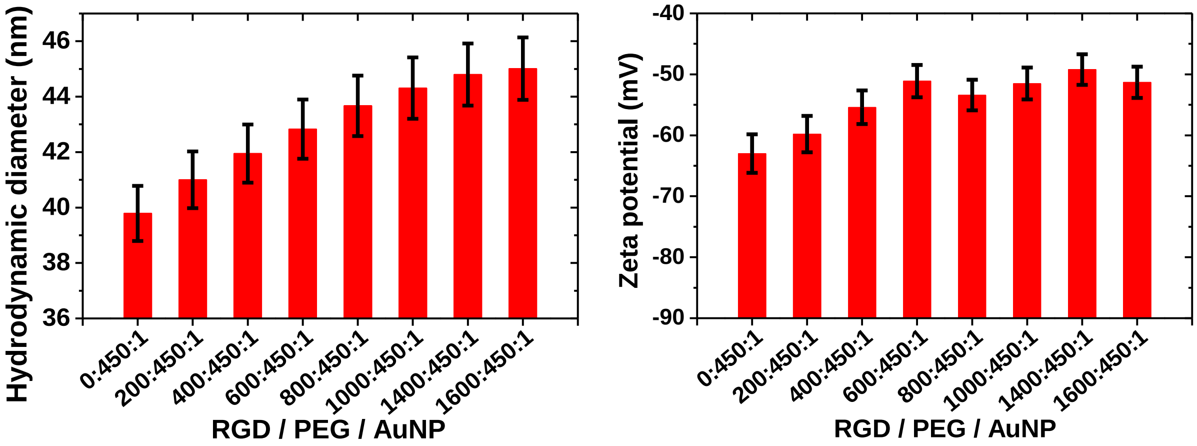

Figure S4. Hydrodynamic diameters (left) and zeta potentials (right) of the AuNP conjugated with different amounts of RGD peptides (the second step of the sequential conjugation). The results on the left show the size of the nanoparticle increases along with the molar ratios of RGD to AuNP (RGD/AuNP). The results on the right indicate that zeta potential of the AuNP first increases with RGD/AuNP and then plateaus for higher molar ratios.

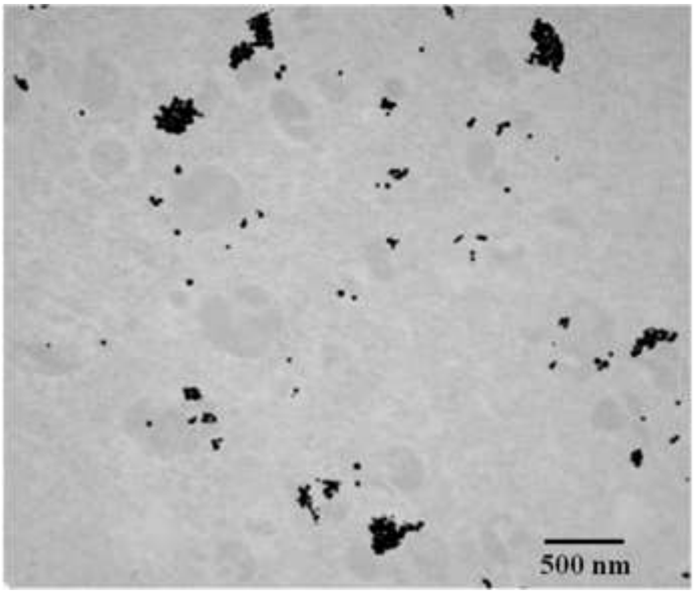

Figure S5. Transmission electron microscopy (TEM) micrograph of intracellular AuNPs (black dots). The result shows that the AuNPs exist and partially aggregate (the clusters) in the endocytotic vesicles inside the cytoplasm.

Table S1. Average radioactivity in media, PBS, and cells at different RGD densities for five measurements.

RGD	Media (unit: CPM[a])	PBS (unit: CPM)	Cells (unit: CPM)	Percentage in cells (%)
200	3042176	58194	11644	0.37
400	4974681	157340	49194	0.95
600	4519522	152054	40763	0.87
800	5276641	224095	79703	1.43
1000	4889383	197239	102211	1.97
1400	5121002	163095	164324	3.02
1600	5150100	207906	200316	3.60

[a] Count Per Minute

Supporting Notes

Note S1: SNR and CNR

Reconstructed photoacoustic microscopy (PAM) images were quantified using both signal-to-noise ratio (SNR) and contrast-to-noise ratio (CNR). The SNR in decibel (dB) is defined as

$$\mathrm{SNR_{dB}} = 20\log_{10}\left(\frac{\mu_{\mathrm{sig}}}{\sigma_{\mathrm{bg}}}\right), \tag{S1}$$

where μ_{sig} is the mean value of the signal, σ_{bg} is the standard deviation of the background. The CNR in dB is defined as

$$\mathrm{CNR_{dB}} = 20\log_{10}\left(\frac{|\mu_{\mathrm{sig}} - \mu_{\mathrm{bg}}|}{\sigma_{\mathrm{bg}}}\right), \tag{S2}$$

where μ_{bg} is the mean value of the background. Table S2 summarizes the SNRs and CNRs of all PAM images in Figure 4 in the paper.

Table S2. Summary of SNRs and CNRs of Figure 4 in the paper.

	**Figure 4(d)**	**Figure 4(e)**	**Figure 4(f)**
SNR (dB)	25.9 (21.7[a])	24.8 (21.4)	27.3 (24.5)
CNR (dB)	23.2 (17.4)	21.7 (17.0)	24.7 (20.6)

[a] for enlarged images

Note S2: Lateral Resolution Calibration of the PAM System

Lateral resolution of the photoacoustic microscopy (PAM) system was calibrated by evaluating the edge spread function (ESF) measured from chrome (Cr) stripes (linewidth: 5 µm, period: 40 µm) coated on a glass slide. The two-dimensional (2D) bright-field and PAM images of the Cr

pattern are shown in Figures S6A and S6B. Based on the assumption that the laser beam profile was Gaussian, the ESF was estimated by averaging the edge of the measured Cr line in the red rectangle, and was fitted using the Gauss error function (R^2 = 0.9991, Figure S6C), which is defined as

$$y = A\,\mathrm{erf}\left(\frac{x - x_0}{\sigma\sqrt{2}}\right) + B, \tag{S3}$$

where A is the amplitude, B is the offset, x_0 is the position of the edge, σ is the standard deviation, and erf is the Gauss error function. The derivative of the ESF is the line spread function (LSF), which is expressed as

$$y' = \frac{2}{\sqrt{\pi}} A\,\mathrm{e}^{-\frac{(x - x_0)^2}{2\sigma^2}}. \tag{S4}$$

The lateral resolution r, defined as the full-width at half-maximum (FWHM) of the LSF, is given as

$$r = 2\sqrt{2\ln 2} \times \sigma. \tag{S5}$$

In this case, the estimated parameters are A = 0.50, B = 0.50, σ = 0.66, x_0 = 4.04. Therefore, the quantified lateral resolution is r = 1.6 μm (see Figure S6C), which is close to the theoretical resolution 0.51λ/NA = 1.4 μm (λ = 0.532 μm, NA = 0.2) determined by the diffraction limit.

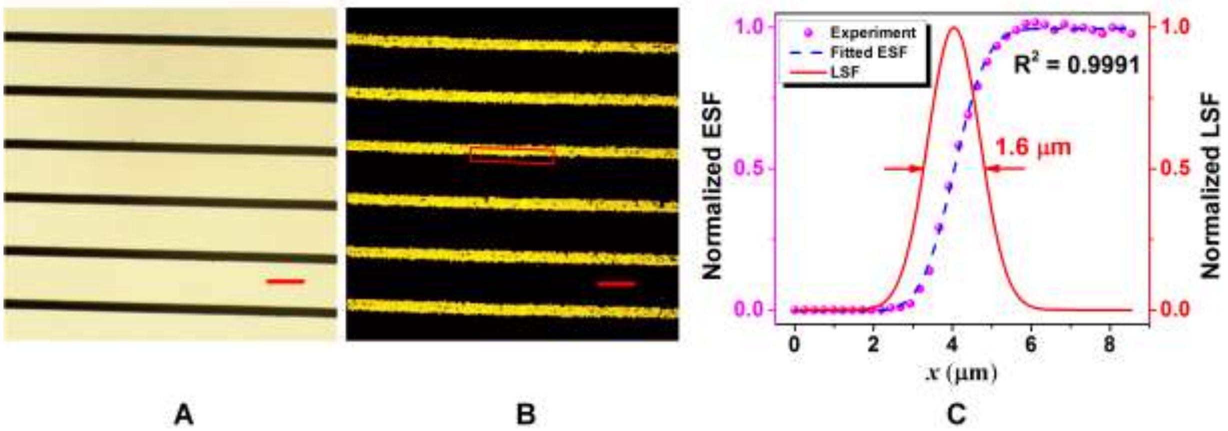

Figure S6. Lateral resolution calibration of the PAM system by imaging a Cr pattern with periodic stripes (linewidth: 5 μm, period: 40 μm). (**A**) Bright-field microscopy image. (**B**) PAM image. (**C**) Measured lateral profile, fitted ESF and LSF of the Cr edge in the red rectangle in (**B**). The calibrated lateral resolution (FWHM) is 1.6 μm. Scale bar: 25 μm.

To further demonstrate the performance of the PAM system, human red blood cells (RBCs, diluted 5000-fold using 1X PBS) from a volunteer donor were imaged. Single RBCs (mean diameter 7.2 μm[1]) were clearly visible in the images (Figure S7).

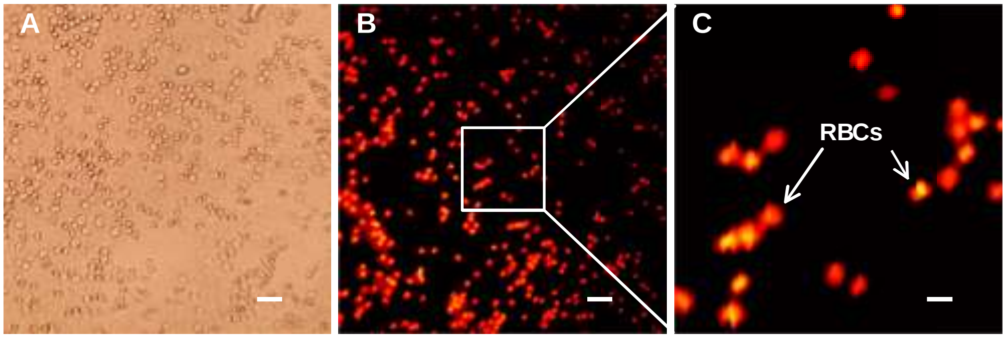

Figure S7. Micrographs of RBCs. (**A**) Bright-field microscopy image. (**B**) and (**C**) PAM images at different magnifications showing single RBCs are photoacoustically visible and discernible. Scale bar: 30 μm for (**A**) and (**B**), 7.5 μm for (**C**).

References

[1] M. Louise, *Clinical Hematology: Theory and Procedures*. 5th ed.; Lippincott Williams & Wilkins: Philadelphia, **2012**.
